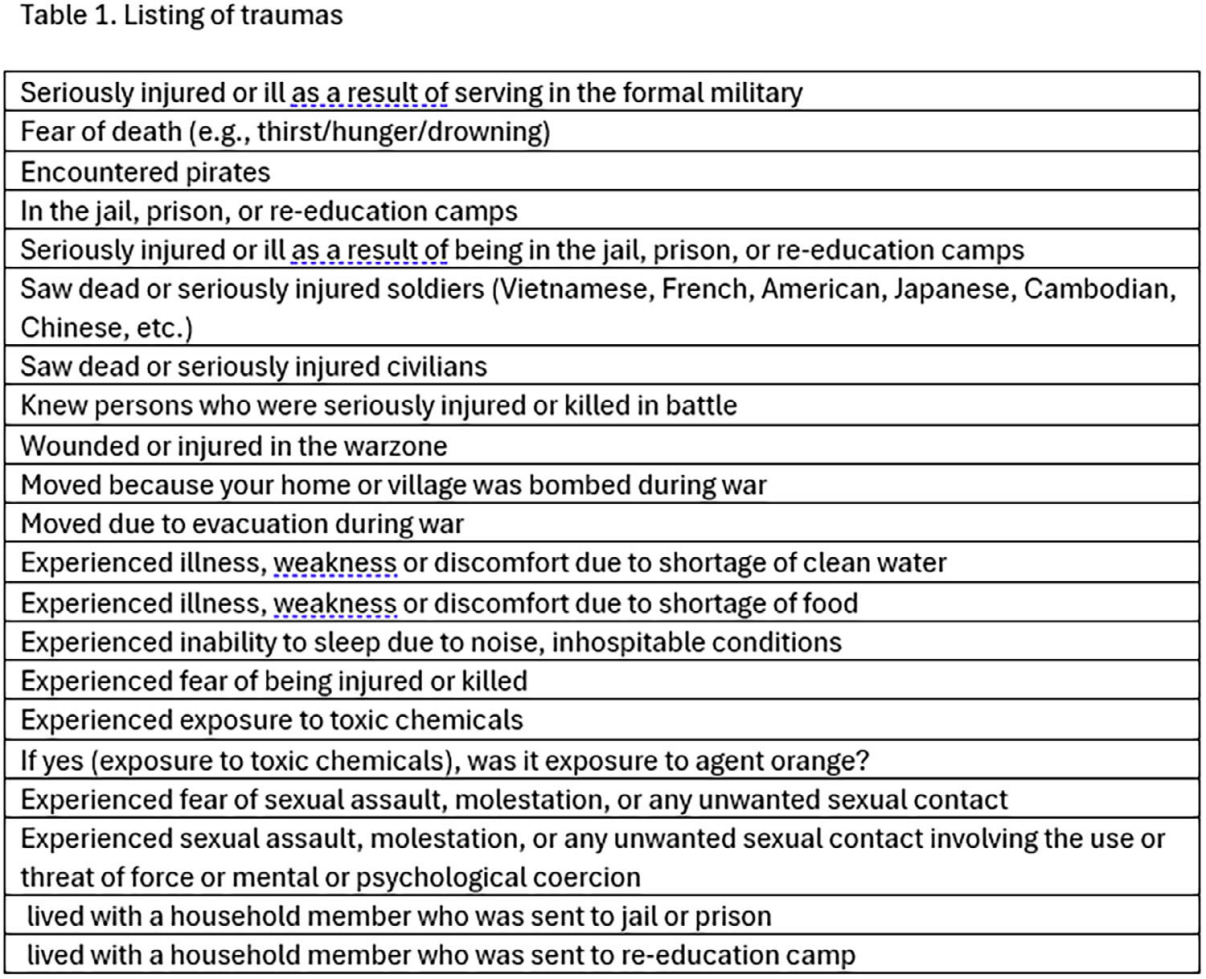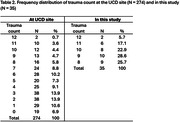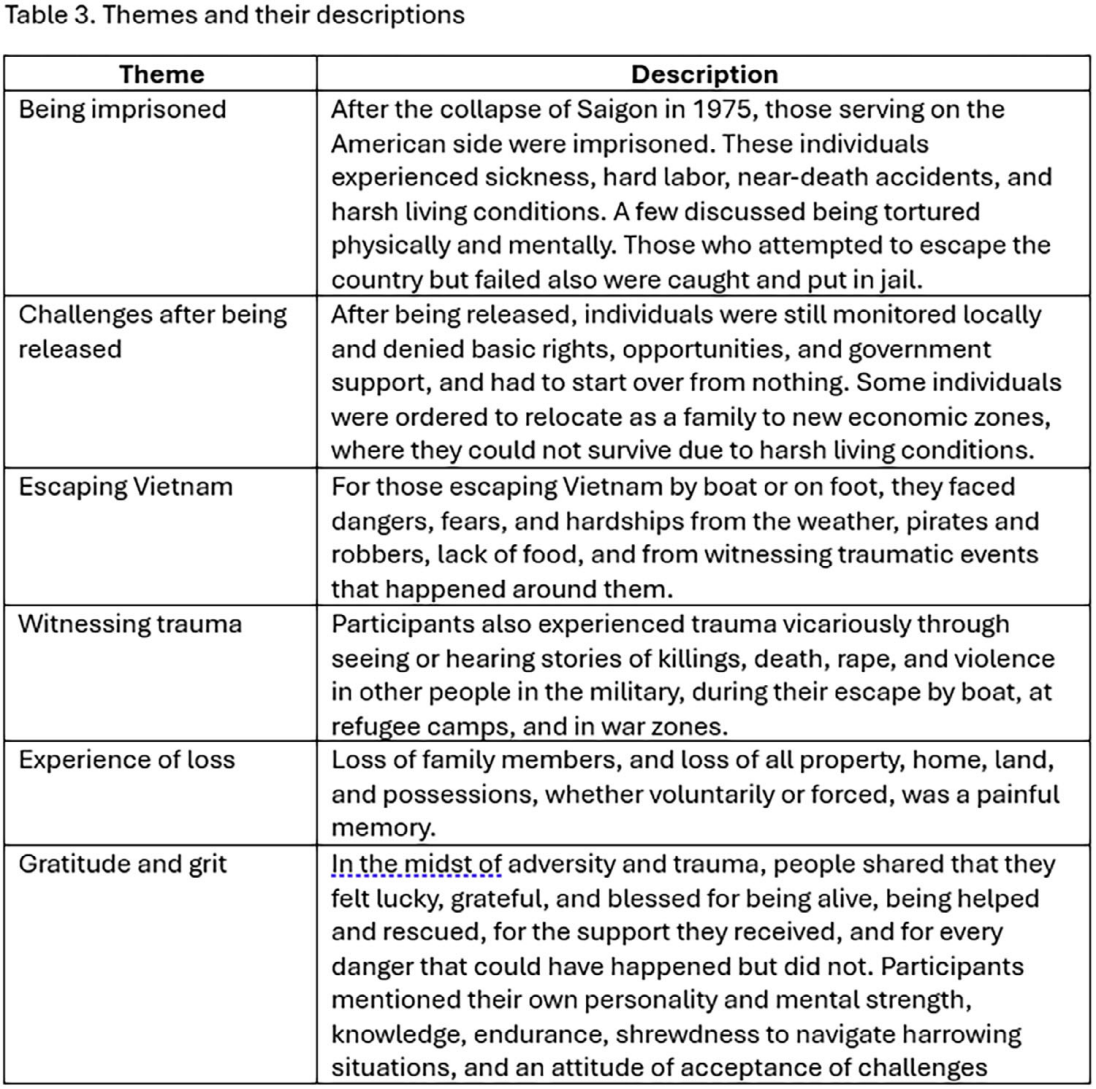# Stories of Trauma and Strength Among Aging Vietnamese Americans: Qualitative Findings from the Vietnamese Insights into Cognitive Aging Program

**DOI:** 10.1002/alz.091067

**Published:** 2025-01-09

**Authors:** Uyen TM Vu, Sarah Tomaszewski Farias, Ladson Hinton, Van Ta Park, Quyen Q Tiet, Rachel A. Whitmer, Oanh L. Meyer

**Affiliations:** ^1^ University of California, Davis School of Medicine, Sacramento, CA USA; ^2^ Multi‐Ethnic Health Equity Research Center, University of California, San Francisco, San Francisco, CA USA; ^3^ VA Palo Alto Health Care System, Menlo Park, CA USA

## Abstract

**Background:**

Previous research demonstrates that stress and trauma are associated with poor health and increased dementia risk, but this is mostly based on studies of non‐Hispanic Whites. This descriptive analysis delineated the war‐related adversity and trauma in participants of the Vietnamese Insights into Cognitive Aging Program (VIP) study, a new cohort of cognitive aging in Vietnamese individuals in Northern California.

**Method:**

VIP is a longitudinal study of 548 Vietnamese Americans aged 65+ years living in Northern California who are seen annually at either research site for three years. During visit 1, a short (15‐20 minutes) semi‐structured interview was conducted with all consented participants about their life and experiences in Vietnam and their immigration to the United States. Using the early life trauma data collected at visit 1 at the Sacramento site, we calculated a total trauma exposure count for all 274 participants and selected out individuals with the highest self‐reported traumas (i.e., top one‐third, see Table 1 for listing of traumas and Table 2 for the frequency distribution of total traumas). We then pulled out the interview data for participants who had consented to be audio‐recorded, whose recordings were of good quality, and whose interviews had been transcribed verbatim. A total of 35 interviews were included in this analysis. Six transcripts were coded independently by two authors until agreement was reached. A codebook was developed, and the remaining transcripts coded and analyzed by the lead author.

**Result:**

The 35 participants had a mean age of 73 years (SD = 5.57). Most were male (71.4%). Forty percent finished high school; 34% had at least a primary school education, and 26% had at least some college. We found six major themes: (1) being imprisoned, (2) challenges after being released, (3) escaping Vietnam, (4) witnessing trauma, (5) experiences of loss, and (6) gratitude and grit (see Table 3).

**Conclusion:**

Results from this study reveal traumatic events 35 individuals in the VIP at the Sacramento site were exposed to as well as their perseverance. Findings provide potential to explain the relationship between trauma/adversity and cognition and dementia risk that will be explored in future VIP research.